# Eicosapentaenoic acid prevents atrial electrocardiographic impairments and atrial fibrillation in high fat diet mice

**DOI:** 10.1016/j.jphyss.2025.100014

**Published:** 2025-03-06

**Authors:** Kosuke Horii, Katsushige Ono, Tomoko Sumi, Mayo Higashihara, Nobuhiro Zaima, Seiji Masuda, Masaki Morishima

**Affiliations:** a Department of Applied Biological Chemistry, Graduate School of Agriculture, Kindai University, 3327–204 Nakamachi, Nara 631–8505, Japan; b Oita Shimogori Hospital, 1410 Shimogori, Oita 870–0926, Japan; c Department of Applied Biological Chemistry, Graduate School of Agriculture, Kindai University, 3327–204 Nakamachi, Nara 631–8505, Japan; d Department of Food Science and Nutrition, Faculty of Agriculture, Kindai University, 3327–204 Nakamachi, Nara 631–8505, Japan; e Yuasa Experimental farm of Kindai University, 2355–2 Yuasa, Wakayama 643–0004, Japan

**Keywords:** Eicosapentaeonic acid, High-fat diet, Atrial fibrillation, Inflammatory cytokines, Fibrosis

## Abstract

There is growing evidence that eicosapentaenoic acid (EPA) uptake has beneficial effects on various cardiovascular diseases. However, electrophysiological actions of EPA remain poorly documented. To investigate the potential antiarrhythmic effects of EPA, mice were fed a high-fat diet (HFD) or an HFD supplemented with EPA for eight weeks. Electrocardiogram (ECG) recordings in combined with esophageal electrical stimulation revealed that HFD-fed mice exhibited bradycardia, reduced P-wave amplitude, and prolonged P-wave duration. Atrial fibrillation (AF) was induced in 100 % of HFD mice, which was only in 50 % of EPA-supplemented mice with significantly shorter durations. HFD-fed mice showed decreased expression of *Cav1.2*-mRNA, increased expression of *Kv1.5*-mRNA, elevated expression of inflammatory cytokines (*IL-1β, TNF-α,* and *IL-10*), and larger fibrotic area in atrial tissue, which were all reversed by EPA supplementation. These findings suggest that long-term dietary intake of EPA may help maintain normal atrial function and structure, thereby reducing the risk of AF.

## Introduction

Atrial fibrillation (AF) is the most common atrial arrhythmia, with the potential to impair cardiac performance and increase the risk of thrombus formation and stroke [1]. Recent clinical studies have demonstrated a significantly higher incidence of AF in patients with dyslipidemia, which suggests that dyslipidemia is a risk factor for the development of arrhythmias [2], [3]. The increasing prevalence of dyslipidemia is attributed to factors such as high-fat, high-sugar, and high-calorie diets, sedentary lifestyles, and heightened social stress [4], [5]. Growing evidence suggests that an HFD can lead to atrial remodeling, which in turn increases susceptibility to AF [6], [7]. In diet-induced obese model mice, atrial muscles exhibit AF as a result of fibrosis and the accelerated formation of AF substrates [6]. However, the precise mechanisms by which electrical remodeling of myocardial ion channels contributes to HFD-mediated AF remain unclear. While several studies have explored the relationship between HFD and AF etiology [8], [9], the molecular mechanisms linking AF to lifestyle-related diseases are still less understood compared to the abundance of clinical evidence. In this context, dietary management is considered an important strategy for preventing this condition. However, previous studies have not reached a consensus on the precise role of dietary factors in AF pathogenesis.

Caffeine, alcohol, HFD or saturated fatty acids, polyunsaturated fatty acids derived from fish, and dietary fiber have been identified as potential dietary factors influencing the development and prevention of AF [4]. Among them, alcohol consumption and the risk of AF has recently been intensively studied [10], [11]. For instance, Larsson demonstrated that an increase in standard daily alcohol consumption is associated with an 8 % elevation in the relative risk of developing AF [10]. A recent study indicates that the transient onset of AF following alcohol consumption is linked to PKC/GSK3β/NFAT signaling in pulmonary vein myocardial cells (PV myocardial sleeve), which leads to the re-expression of T-type Ca²⁺ channel [12]. This electrical remodeling in the pulmonary veins is strongly associated with AF development. The mechanisms by which alcohol consumption increases susceptibility to AF include shortening of the atrial refractory period, attenuation of vagal activity, sympathetic activation, decreased heart rate, and altered atrial excitability. While numerous lines of evidence suggest that HFD-induced AF contributes to atrial electrical and structural remodeling, leading to AF development in dyslipidemia models [7], [8], we currently have quite limited understanding as to the rescue or nutrients which can prevent or ameliorate diet-induced AF.

Some studies have recently highlighted the cardioprotective effects of omega-3 polyunsaturated fatty acids, particularly eicosapentaenoic acid (EPA) and docosahexaenoic acid (DHA) from fish oil, in reducing the risk of arrhythmias and sudden cardiac death [13], [14]. Regular consumption of 1–2 fish meals per week is consistently associated with a reduced risk of cardiovascular disease compared to infrequent or no fish consumption [15]. In our previous study, we demonstrated that EPA reverses the decline in Cav1.2-Ca²⁺ channel function caused by saturated fatty acid-induced lipotoxicity and oxidative stress. This rescue occurs via both free fatty acid receptor 4 (FFAR4)-dependent and -independent pathways in mouse cardiomyocytes. Notably, the electrophysiological effects of EPA on cardiomyocytes appear not to be limited to FFAR4 pathways. The dense distribution of FFAR4 in the atrium suggests that the actions of EPA on Ca²⁺ channels are most likely to occur in atrial tissue [16]. These findings indicate that atrial electrical and structural remodeling associated with HFD-induced dyslipidemia may be mitigated by EPA administration, providing a potential therapeutic strategy for the prevention of AF in diet-induced dyslipidemia models.

This study aimed to investigate whether excess dietary fat affects the atrial myocardium and contributes to the substrate for AF, and whether these effects can be ameliorated by EPA. Specifically, the study examined and compared surface electrocardiograms (ECG), atrial pacing responses, and key proteins potentially involved in cardiac remodeling in mice fed either a normal diet or an HFD with or without EPA supplementation.

## Materials & methods

### Animals

Male C57BL/6 mice (Japan SLC, Inc., Shizuoka, Japan) were provided with food and water ad libitum and the room temperature was maintained at 25 °C ± 1 °C in a 12-hour light/12-hour dark cycle (light phase; 8:00–20:00). The purchased mice were acclimated to the new environment for at least two weeks before experiments. All experiments were performed on male me between 7 and 15 weeks of age. All animal experiments were approved by the Kindai University Animal Care and Use Committee and were performed according to the Kindai University Animal Experimentation Regulations (Approval number: KAAG-2020–015, and KAAG-2023–003) and were carried out according to the guidelines for animal research of the Physiological Society of Japan to minimize the number of animals used as well as their suffering.

### Experimental model of diet-induced dyslipidemia

To evaluate the effect of HFD and/or EPA on AF, we divided into three groups; control (n = 15), HFD (n = 15), HFD+EPA (n = 15) groups. The control mice were given ad libitum access to a regular diet (RD, MF, CLEA, Tokyo, Japan), which has an energy density of 3.6 kcal/g with 61.2 % from carbohydrate, 26 % from protein, and 12.8 % from lipids, combined with drinking distilled water. HFD groups of mice were fed a HFD (High fat diet 32, CLEA, Japan), which has an energy density of 5.1 kcal/g with 19.9 % from carbohydrate, 20.1 % from protein, and 60 % from lipids, combined with 4 % sugar solution (23.2 g/L fructose, 18.8 g/L sucrose; Fujifilm Wako Pure Chemical Industries, Osaka, Japan) for 8 weeks. In accordance with previous reports [17], daily oral administration of EPA (300 mg/kg/day, Mochida Pharmaceutical, Shinjuku, Japan) via gastric tube was continued for 8 weeks. EPA was mixed with phosphate-buffered saline (PBS; Fujifilm Wako, Japan) containing 0.4 % polyoxyethylene (20) sorbitan monolaurate (Fujifilm Wako) and 5.6 % dimethyl sulfoxide (DMSO; Sigma Chemical, St. Louis, MO). Body weight, food consumption, and water consumption were recorded once a week. After in vivo electrophysiological measurements, the animals were sacrificed by exsanguination under deep isoflurane anesthesia by extracting blood from the apex of the heart. Hearts (atria and ventricles) and other tissues were harvested, snap frozen on dry ice, and stored at −30 °C.

### Electrophysiological measurements

After 8 weeks, mice were anesthetized with 1.0–1.5 % isoflurane with 100 % oxygen, and electrodes were attached to the limbs. Three-lead ECG recordings were obtained with BioAmp (AD Instruments, Dunedin, New Zealand) and converted to digital signals for analysis using LabChart (AD Instruments). Only high-quality ECGs were used in this study with high magnification (200–400 %). PR intervals, QRS duration and P wave duration of three consecutive beats were measured by a single technician who was blind to information about the experimental groups, and mean values were used for analysis. All measurements were re-checked by an expert on cardiac physiology.

### Induction of AF

AF in mice was induced by transesophageal electrical burst pacing as described by Suita et al. [18]. Briefly, mice were anesthetized by inhalation of isoflurane (1.5 % for maintenance). A 1.1-F octapolar catheter with eight 0.5-mm circular electrodes and an interelectrode spacing of 1 mm (EPR800; Millar Instruments) was carefully inserted into the esophagus of each mouse. The catheter was fixed at the site where the height of the atrial electrogram on the esophageal electrocardiogram (ECG) was the highest. Transesophageal atrial burst pacing was performed for 10 s at a stimulation amplitude of 1.5 mA with 10-ms cycle lengths and a pulse width of 3 ms while monitoring the lead II body surface ECG. The duration of AF was measured based on its apparent length on the ECG, with AF on the ECG defined according to the following criteria: (i) loss of the P wave, (ii) irregular R-R interval, and (iii) duration greater than 2 s. The stimulation protocol for the experiment to induce AF using atrial high-frequency stimulation is shown below.

**[Protocol 1]** Single trial of burst pacing for the duration of 10 s was performed in individual mice, and the incidence and duration of AF were recorded.

**[Protocol 2]** Three sequential trials of burst pacing for the duration of 10 s were performed in individual mice; the second and the third trials were performed with three minutes’ interval. The longest episode was recorded as the duration of AF.

### Measurement of blood flow and body temperature

After 7 weeks, we measured the blood flow in the lower limbs of mice assessed by OMEGAZONE, OZ-2 mini (Omega Wave Corporation, Tokyo, Japan). Under anesthesia by medetomidine (Orion Oyj, Espoo, Finland), midazolam (Maruishi Pharmaceutical Co., Ltd., Osaka, Japan), and butorphanol (Meiji Animal Health Co., Ltd., Tokyo, Japan), mice were placed on the experimental table so that the camera could capture their toes and heels. Measurements of blood flow in the lower limbs of mice were acquired using the observation application (LSI-U421) supplied with the OMEGAZONE OZ-2 mini under the condition of 100 consecutive images at high resolution (16 bit, 639 ×480 resolution). The image data were used to quantify the fluorescence intensity at the fingertips and heel of the same individual using the accompanying analysis application (LIA-v433) to calculate the blood flow rate. After measuring the blood flow rate, the body surface temperatures of the toes and abdomen of the mice were measured using a thermal imaging camera (FLIR E4: Teledyne FLIR LLC, Oregon, USA).

### Quantitative real-time PCR of atrial tissues

After in vivo electrophysiological measurements, left and right atria were immediately rinsed in PBS, snap frozen in liquid nitrogen, and stored at −30 °C until real-time PCR analysis. Total RNA was extracted from atrial tissue using TRIzol™ reagent (Thermo Scientific, Massachusetts, USA) according to the manufacturer's protocol. Total RNA concentration was determined using a spectrophotometer (NanoDrop Lite Plus; Thermo Scientific). The cDNA was synthesized from 200 ng total RNA using ReverTra Ace® qPCR RT Kit (TOYOBO). Real-time PCR was performed using SYBR Green (THUNDERBIRD® qPCR Mix: TOYOBO) and primers were purchased by Hokkaido System Science (Hokkaido system sciences, Sapporo, Japan) (Table 1).

**Table 1:** Primer sequences used for real-time PCR.

**Target (gene name)**	**Gene Bank accession No.**	**Primer sequences**
*GAPDH*	GU214026	F: 5′-CCACCCAGAAGACTGTGGAT−3′ R: 5′- CACATTGGGGGTAGGAACAC−3′
*Nav1.5 (SCN5A)*	NM_001253860	F: 5′-CTTCACCAACAGCTGGAACA−3′ R: 5′-GACATCATGAGGGCGAACAG−3′
*Cav1.2 (CACNA1C)*	NM_001255999	F: 5′-ACATCTTCGTGGGTTTCGTC−3′ R: 5′-TGTTGAGCAGGATGAGAACG−3′
*Kv1.5 (KCNA5)*	NM_145983	F: 5′-TATCATCGGGAGACAGACCAC−3′ R: 5′-CCAGACAGAGGGCATACAGAG−3′
*Cx40*	NM_001271628	F: 5′-ATTCTGATCCGCACCACCAT−3′ R: 5′-CATGCAGGGTATCCAGGAAGA−3′
*IL−1 β*	NM_008361	F: 5′-GCAACTGTTCCTGAACTCAACT−3′ R: 5′-ATCTTTTGGGGTCCGTCAACT−3′
*TNF-α*	NM_013693	F: 5′-ATGAGCACAGAAAGCATGA−3′ R: 5′-AGTAGACAGAAGAGCGTGGT−3′
*IL−10*	NM_010548	F: 5′-GGTGAGAAGCTGAAGACCCT−3′ R: 5′-ACACCTTGGTCTTGGAGCTT−3′
*Col1a1*	NM_007742	F: 5′-TGAACGTGGTGTACAAGGTC−3′ R: 5′-CCATCTTTACCAGGAGAACCAT−3′

Glyceraldehydes-3-phosphate dehydrogenase (*GAPDH*; GU214026) mRNA was used as an internal control. Data were calculated by 2^−∆∆CT^ and presented as fold change in transcripts for target genes in myocytes and normalized to *GAPDH* (defined as 1.0 fold).

### Histology

Histologic evaluation of the left atrium was performed using Masson's trichrome staining. Isolated left atria were fixed in 4 % paraformaldehyde (Fujifilm Wako, Japan), embedded in paraffin, and sectioned at 5 µm. Masson's trichrome staining was used to evaluate interstitial fibrosis. Masson trichrome staining was subcontracted to the Tokushima Institute of Molecular Pathology (Tokushima, Japan). Images were captured and digitized on a BIOLEVO BZ-9000 epifluorescence microscope (Keyence, Osaka, Japan) and analyzed at 400 × magnification using the associated software (Keyence), and the area of fibrosis was analyzed using Image J ver.1.53 (Wayne Rasband, National Institutes of Health, Bethesda, MD, USA). In each atrium, four images were analyzed at × 400 magnification and averaged (the number of mice in each group was three).

### Statistical analysis

All experimental data are expressed as mean ± standard error (SE). Comparisons between groups were tested for normality (Kolmogorov-Smirnov-test) according to EZR ver. 1.61, followed by a test of equal variance (Bartlett-test) if normality was found. Group data for which equal variances were found were checked for significance by one-way ANOVA and then subjected to multiple comparisons using the Tukey-Kramer method. Data for which normality was not found were checked for significance by non-parametric tests (Kruskal-Wallis-test) and then subjected to multiple comparisons by the Mann-Whitney U-test and Steel-Dwass. The significance level for all statistical treatments was set at less than 5 % (p < 0.05).

## Results

### HFD induced dyslipidemia

**Fig. 1:**
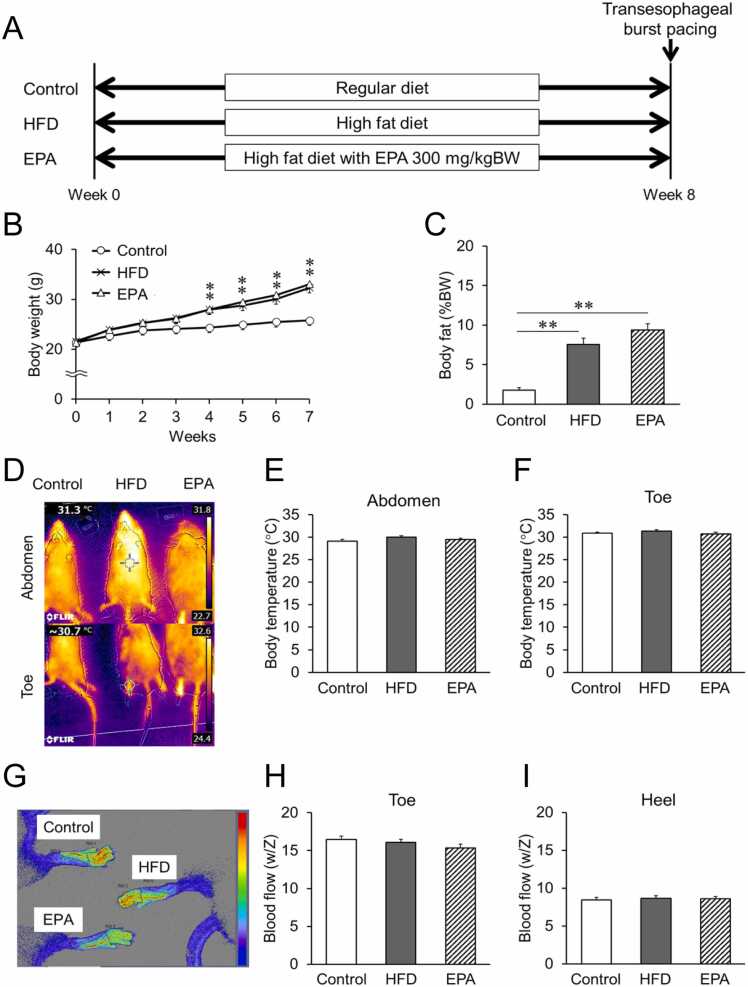
Effects of HFD on systemic metabolism in mice, (A) Protocol for the HFD mouse model, which included feeding with HFD and drinking a 4 % sugar solution (23.2 g/L fructose and 18.8 g/L sucrose). (B) Changes in body weight over 8 weeks. (C) Body fat mass (%) at the time of dissection. (D) A representative thermographic image showing body temperature. The top row represents the abdomen, and the bottom row represents the toes of mice. (E) Body temperature of the abdomen and (F) toes, calculated from the captured images. (G) A typical image used to evaluate blood flow in a mouse, obtained with the OMEGAZONE OZ-2 mini. Blood flow of the toe (H) and heel (I) of each mouse from the captured images. Data are expressed as mean ± SE (n = 15). *p < 0.05, * *p < 0.01, by Kruskal-Wallis test with Steel-Dwass multiple comparison.

To investigate whether differences in diet quality influence the incidence and duration of AF and whether EPA has a preventive effect, we developed a mouse model fed either a normal diet or an HFD for 8 weeks (Fig. 1A). Physical and cardiac functional changes induced by the diets were evaluated. Compared with control mice, those fed the HFD exhibited greater weight gain over time, reaching statistical significance at 4 weeks and persisting until the end of the experiment at 8 weeks (Fig. 1B). At the conclusion of the study, HFD-fed mice had significantly more visceral fat compared to the control group (Fig. 1C). Given the hypothesis that long-term HFD consumption could affect body temperature, thermographic measurements were performed. However, no changes in body temperature were observed in any group, regardless of HFD with EPA consumption (Fig. 1D-F). Blood flow in the lower limbs (toes and heels) was also examined, as EPA has been reported to improve blood flow (Fig. 1G-I). Despite this, the diet did not significantly impact blood flow in the toes or heels of mice in any group at the condition employed in this study.

### Effects of HFD and/or EPA on electrophysiological parameters and susceptibility to AF

**Fig. 2:**
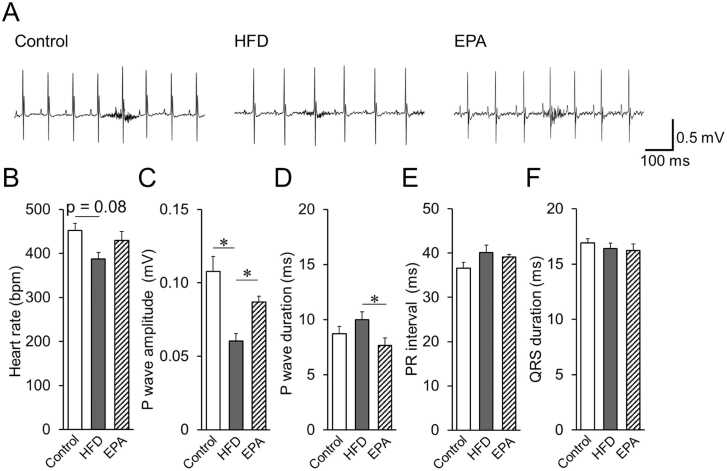
Analysis of surface ECG in each group of mice, (A) A representative trace of the surface ECG in the Control, HFD, and EPA groups. (B) Heart rate calculated from surface ECG recordings at rest. Surface ECG parameters in each group of mice (n = 6). P-wave amplitude (C), P-wave duration (D), PR interval (E), and QRS duration (F) are indicated. Data are expressed as mean ± SE (n = 6). *p < 0.05, by Kruskal-Wallis test with Steel-Dwass multiple comparison.

**Fig. 3:**
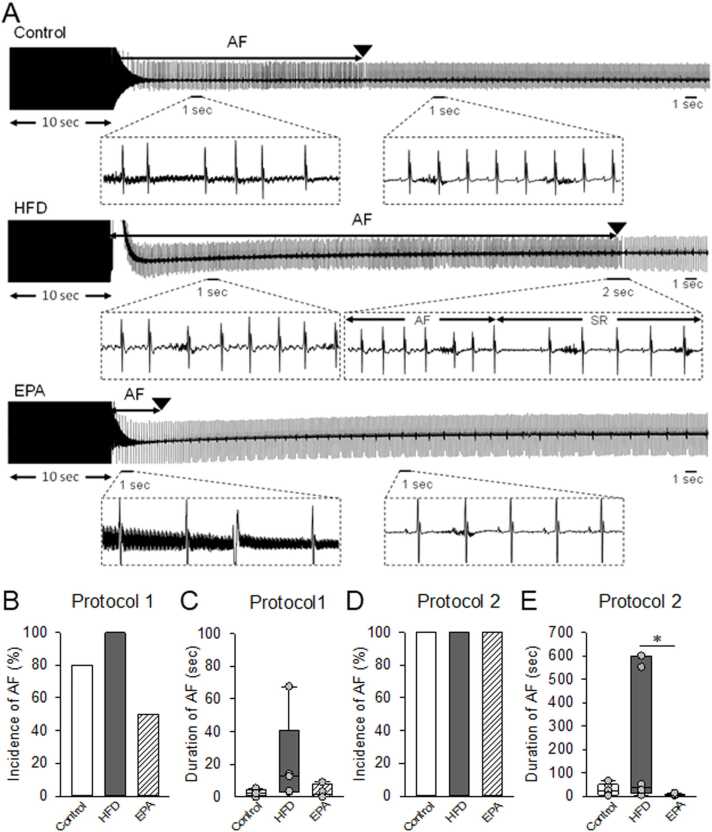
Induction of AF by transesophageal burst pacing, (A) Transesophageal burst pacing. Representative AF induction in mice. Two protocols were examined for each group of mice: [Protocol 1] A single trial of burst pacing was performed in individual mice to induce AF, and the incidence (B) and duration (C) of AF were recorded. AF was invariably induced in Control or HFD mice, whereas it was less frequently induced in EPA mice. Scatter plot data are expressed as median ± SE (n = 5). Black lines indicate median values. [Protocol 2] Three trials of burst pacing were performed in individual mice to induce AF in 100 % of the mice (D). Three minutes after returning to sinus rhythm (SR), the next pacing was performed, and the longest episode was recorded as the duration of AF (E). Scatter plot data are expressed as median ± SE (n = 6). Black lines indicate median values. *p < 0.05, by One-way ANOVA with Tukey's multiple comparison test.

To assess cardiac electrophysiological properties, surface electrocardiograms (ECG) were recorded after 8 weeks of feeding with an HFD or a regular diet (control) (Fig. 2A). In the HFD group, the RR interval was prolonged at rest, indicating a tendency toward bradycardia (Fig. 2B). The amplitude of the P wave was significantly reduced in HFD-fed mice (Fig. 2C), and the duration of the P wave was notably longer in HFD mice compared to EPA-treated mice (10.0 ± 1.7 ms vs. 7.7 ± 1.3 ms) (Fig. 2D). However, no differences were observed among the three groups in the PR interval (Fig. 2E) or QRS duration (Fig. 2F). Furthermore, there was no significant difference between these groups when we have analyzed R-wave amplitudes in the group data (data not shown). Next, we evaluated the incidence and duration of AF in mice under isoflurane anesthesia during a single high-frequency transesophageal atrial stimulation (Fig. 3A). Esophageal delivery of rapid burst stimulation triggered episodes of AF, which occurred with greater frequency and for a longer duration in HFD-fed mice compared to controls (Fig. 3B, C). In contrast, concurrent administration of EPA reversed these effects. As shown in Fig. 3D and E (protocol 2), transesophageal atrial high-frequency stimulation was performed three times, and AF duration and ECG parameters were analyzed when AF incidence reached 100 % in all mice (Fig. 3D). The duration of AF following high-frequency atrial pacing was significantly prolonged in the HFD group. However, these changes were reversed in the EPA-treated group (Fig. 3E). In summary, these results demonstrate that long-term HFD feeding induces cardiac remodeling and increases susceptibility to AF, consistent with previous reports [8]. Importantly, EPA administration effectively rescues these pathological changes in mice.

### Effect of HFD on electrical remodeling in the atria

**Fig. 4:**
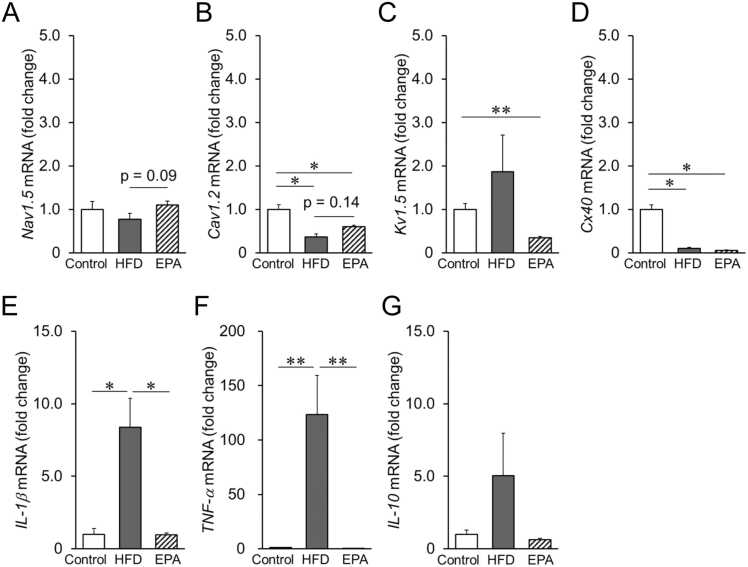
Expression of ion channels and inflammatory cytokines in mouse atria, The effect on electrical remodeling was assessed by ion channel mRNA expression levels in the atria using real-time PCR. The mRNA expression levels of ion channels related to electrical remodeling, including Nav1.5 (A), Cav1.2 (B), Kv1.5 (C), and Cx40 (D) in the atrium after feeding HFD with or without EPA for 8 weeks, are shown. The mRNA expression levels of inflammatory cytokines IL-1β (E), TNF-α (F), and IL-10 (G) in the atrium are also presented. The amount of each mRNA was normalized to that of the control, which was assigned a value of 1.0. Data are expressed as mean ± SE (n = 4–6). *p < 0.05, * *p < 0.01, by Kruskal-Wallis with Steel-Dwass multiple comparison test.

To investigate the mechanisms underlying the observed electrophysiological changes, the mRNA expression levels of critical ion channels were analyzed in the left atrium. As shown in Fig. 4A, the expression level of the voltage-gated Na⁺ channel (*Nav1.5*)-mRNA, which regulates atrial conduction velocity (CV), tended to decrease in HFD-fed mice but was restored by EPA treatment. Similarly, the L-type Ca²⁺ channel (*Cav1.2*)-mRNA, which is essential for atrioventricular conduction, was significantly reduced in HFD mice (Fig. 4B), while the voltage-gated K⁺ channel (*Kv1.5*)-mRNA, a key potassium channel involved in atrial repolarization, was increased compared to control mice (Fig. 4C). These changes were reversed following EPA administration. In contrast, the HFD-induced reduction in connexin 40 (*Cx40*)-mRNA expression was not significantly reversed by EPA (Fig. 4D). These findings indicate that HFD induces electrical remodeling by altering the expression of ion channels in atrial myocytes, whereas EPA exhibits a preventive effect against these changes except the expression of *Cx40*-mRNA.

### Effect of HFD on atrial inflammation and fibrosis in mice

**Fig. 5:**
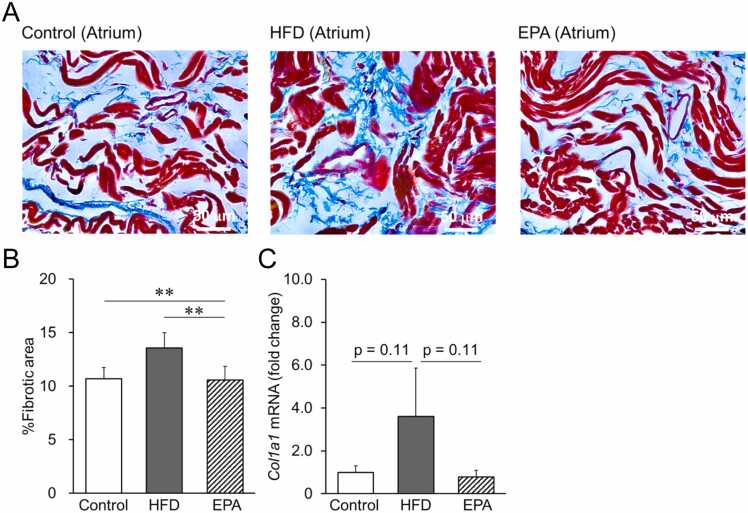
Analysis of atrial fibrosis in mice, Atrial fibrosis in the left atrium was evaluated by Masson’s trichrome staining. (A) Representative images of Masson’s trichrome staining in the atrium of mice. The blue area indicates collagen deposition, and the red area indicates muscle tissue in each group. Scale bars represent 50 µm. (B) Quantification of the fibrotic area in the atrium (%). (C) The mRNA expression of Col1a1 in the atrium. Data are expressed as mean ± SE (n = 4). *p < 0.05, by Kruskal-Wallis with Steel-Dwass multiple comparison test.

Dyslipidemia is known to be associated with chronic low-grade inflammation [19]. We hypothesized that fibrosis resulting from chronic inflammation could reduce CV in the atrium, thereby increasing the inducibility of AF in HFD-fed mice. To investigate the extent of inflammation, cytokine levels in the atria were quantified using real-time PCR. The expression of both interleukin (*IL)−1β*- and tumor necrosis factor-α (*TNF-α*)-mRNA was significantly increased in the atria of HFD mice compared to control mice (Fig. 4E, F). *IL-10*-mRNA expression was also upregulated in the atria of HFD mice (Fig. 4G), although this change did not reach statistical significance. We confirmed that long-term HFD treatment significantly induced atrial inflammation, which was reversed by co-administration of EPA (Fig. 4). Additionally, histological analysis revealed increased atrial fibrosis in HFD-fed mice compared to control mice, as evidenced by blue staining, which indicates interstitial fibrosis (Fig. 5A). The quantitative ratio of the fibrotic area to the reference tissue area is summarized in Fig. 5B. As shown in Fig. 5C, *Col1a1* mRNA expression analysis revealed upregulated interstitial atrial fibrosis in HFD mice, which was not observed in EPA-treated mice. These results suggest that chronic inflammation and atrial fibrosis caused by long-term HFD feeding may contribute to the persistence of AF by increasing the vulnerability to AF. Importantly, EPA may prevent these adverse effects.

## Discussion

The present study provides the first evidence that EPA, a type of polyunsaturated fatty acid (PUFA), prevents AF in a well-established HFD mouse model. The main findings of this study are as follows: (1) long-term treatment with HFD for 8 weeks induced structural remodeling, including abnormal expression of ion channels and atrial fibrosis in the left atrium; (2) HFD increased the incidence and duration of AF induced by high-frequency atrial pacing; (3) co-administration of EPA at 300 mg/kg body weight (BW) per day prevented the development of AF by inhibiting HFD-induced inflammation and associated pathophysiological changes, thereby preserving atrial excitability and conduction. However, despite the effective concentration of EPA (300 mg/day) in preventing AF, no systemic metabolic benefits were observed, including improvements in perfusion or lipid metabolism. Additionally, EPA did not prevent the increase in body weight or body fat induced by HFD. Instead, EPA appeared to suppress inflammation, prevent myocardial fibrosis, and mitigate conduction disturbances between the right and left atria. These effects seem to occur through a mechanism independent of metabolic improvements. Based on these findings, it is suggested that long-term dietary intake of EPA may serve as a new nutrient contributing to the primary prevention of AF by reducing the increased atrial vulnerability caused by HFD and maintaining normal electrical activity.

### Dyslipidemia and AF

Dyslipidemia is suggested to be an important contributor risk for the development of AF, which may cause the transition from paroxysmal to persistent AF [5]. Also obesity is a major risk factor for AF [5]. It is indeed well known that weight loss can reduce the risk of developing AF, as obesity is a modifiable risk factor that can be influenced by diet. Wong and colleagues reported that the rate of AF after surgery increased by 10 % for each 5-unit increase in BMI, and that weight loss was associated with improvements in both the rate and chronicity of AF [20]. Individual differences in body size have also been shown to correlate with the incidence of AF, with adults who have higher body height, BMI, and lower heart rate being at increased risk for AF [4], [20]. However, in the present study, EPA intake did not affect the weight gain process in HFD-fed mice, which may importantly suggest that the adverse effects of HFD occur independently of obesity. This study aimed to examine whether an excess of dietary fat directly impacts the atrial myocardium and contributes to the formation of an AF substrate. Under atrial burst pacing with the protocol 1 conditions, the heart rate tended to decrease, and the incidence of AF was 100 %. In addition to increased AF susceptibility, some mice exhibited AF durations exceeding 10 min (600 s). In contrast, co-administration of 300 mg/kg/day EPA had no effect on the heart rate but significantly reduced the incidence and duration of AF. Notably, EPA did not change body temperature, nor did it enhance blood flow in obese mice, suggesting that EPA may suppress AF induced by HFD through a mechanism independent of lipid metabolism.

An interesting question that remains to be addressed is why HFD causes atria-specific remodeling, leading to the development of AF. Although underlying inflammatory atrial cardiomyopathy, seen in AF, may contribute to its development, the arrhythmogenic remodeling caused by atrial inflammation has not yet been fully elucidated. Clinical evidence suggests that the prevalence and prognosis of AF are associated with serum levels of inflammatory biomarkers, such as IL-6, C-reactive protein (CRP), TNF-α, and monocyte chemoattractant protein (MCP)−1 [21]. In our study, HFD-induced changes in atrial characteristics and atrial inflammation were already evident after 8 weeks. Atrial-specific changes in the expression of inflammatory cytokines, which likely contribute to atrial-specific remodeling, were also demonstrated in a recent study of HFD-fed rats [22], [23]. Although local inflammation may promote electrical and/or structural remodeling of the atria, increasing susceptibility to AF, these changes were not observed in the ventricle in this study. This raises the question: why does HFD induce these changes specifically in the atrium but not in the ventricle? Our hypothesis is that HFD and dyslipidemia lead to increased body mass and altered renal function, resulting in volume overload [9]. Since the atria are more sensitive to volume overload, they undergo electrophysiological and structural remodeling before the ventricles. Moreover, another important hypothesis is that the free fatty acid receptor 4 (FFAR4), a G protein-coupled receptor for endogenous medium- or long-chain fatty acids that attenuates metabolic diseases and inflammation, may play a role.

The localization of fatty acid receptors in the atria and ventricles may influence their effects. PUFAs, such as EPA, are full agonists of FFAR4 [24], [25], which is expressed in several cell types, including cardiomyocytes [26]. Our previous study showed that FFAR4 expression in the atrium was 25 times higher than in the ventricle, suggesting that the activation of FFAR4 in the atrium could influence pacemaker rhythms [16]. In fact, the amplitude of the P wave was significantly reduced in HFD mice, and the duration of the P wave was significantly longer in HFD mice compared with EPA mice. In contrast, the PR interval and QRS duration did not differ among the three groups. Consistent with these findings, electrical remodeling, inflammation, and fibrosis in the ventricle were not altered by HFD compared to the atrium in this study. It is possible that a new metabolic and lipidomic status associated with HFD leads to structural and electrical remodeling of the atria, thereby contributing to AF vulnerability.

### Protective effect of EPA on the electrical remodeling of HFD induced AF

Prolonged AF duration due to inflammation reportedly results in shortening of the atrial refractory period, abnormalities in the expression of Nav1.5, Cav1.2, and Kv1.5, leading to the progression of electrical remodeling [27] accompanied with structural remodeling, including atrial enlargement and fibrosis, occurs [28]. In this study, the expression of Nav1.5 and Cav1.2 mRNA consistently decreased in the left atrium of the HFD group, while the expression of Kv1.5 mRNA increased. These changes suggest that electrical remodeling occurred, potentially causing conduction disturbances in the atrium. Indeed, downregulation of Nav1.5 and Cav1.2 mRNA and protein expression, upregulation of Kv1.5 mRNA, along with conduction defects and fibrosis, have been observed in the atria of both AF patients and experimental obese animal models in agreement with our results [29]. Of particular note is the decrease in the expression of Nav1.5 mRNA and the increase in Kv1.5 mRNA observed in the HFD group. Persistent AF causes the loss of atrial myocytes and the progression of fibrosis, resulting in delayed atrial conduction and the formation of a re-entry circuit, which increases the likelihood of AF [30]. Since Na⁺ channel remodeling delays atrial conduction, the lack of change in Nav1.5 mRNA expression with co-administration of EPA is consistent with the results of P-wave analysis. Thus, EPA could help maintain the excitability of the atria and support conduction from the right to the left atrium.

In addition, mRNA expression of the Kv1.5 gene is known to increase in response to myocardial stretch, radiofrequency excitation, and alcohol challenge [31]. In the present study, an increase in Kv1.5 mRNA expression was observed in the HFD group compared with the control group, suggesting that the sustained atrial stretch induced by dyslipidemia and/or obesity resulted in an increase in Kv1.5 mRNA expression. The expression and enhanced function of Kv1.5 channels shorten the action potential duration of the atria, thereby shortening the refractory period. Importantly, co-administration of EPA reversed the level of Kv1.5 mRNA expression. This suggests that EPA may act as a nutrient with a preventive effect on atrial stress. Although we do not have sufficient data on the molecular mechanism for this ion channel transcription by EPA, we speculated that EPA could act directly and indirectly on the ion channel remodeling. Both intracellular Ca^2+^ dependent protein kinases (protein kinase-α or -δ, calmodulin kinase) and oxidative stress induced by HFD has been strongly suggested a cause-effect relationship between ion channel remodeling and cardiac excitability [16], [32]. Of note, previous reports have shown that abnormal cardiac function caused by an intracellular Ca^2+^ overload and reactive oxygen scavenger (ROS) accumulation are affected by dyslipidemia and that EPA rescues both via its receptor FFAR4-dependent or -independent pathways [16], [26]. Consequently, we hypothesized that, in addition to the direct inhibitory effect of EPA on the aberrant expression of ion channels induced by a HFD, EPA alone may regulate ion channel expression. However, since the blood EPA concentration in the mice used in this experiment was not measured in this study, this question remains unresolved, and future studies are needed. Furthermore, the reduction in Cx40 mRNA expression in the HFD group was not rescued by EPA. Regarding the short half-life of this type protein [33], a period of 8 weeks appears to be long enough for the observed changes to occur. Further studies are obviously needed to explore these questions.

### Long-term antiarrhythmic effects of EPA

To more closely mimic clinical situations, it is important to study the effects of dietary EPA supplementation in animal models after long-term (weeks to months) feeding. Clinically, the protective effects of EPA supplementation typically lag by several months after starting EPA [34], so changes in gene expression must be considered. Prolonged EPA supplementation may affect membrane protein function through at least three mutually exclusive mechanisms. First, the membrane material properties could be altered by the incorporation of polyunsaturated acyl chains of EPA into membrane phospholipids [35]. Second, increasing EPA levels in the membrane lipid bilayer may facilitate phase separation such as lipid rafts, leading to altered function and modulation of membrane protein-associated lipid rafts [36]. Third, EPA can modulate gene expression through the regulation of the activity of several transcription factors [16], [37]. Therefore, to discuss the molecular mechanism of the antiarrhythmic effect of EPA, it is necessary to formulate a working hypothesis based on the effect of EPA as a lipid on cells.

Another important finding of our study is that prolonged dietary EPA supplementation in mouse models resulted in an antiarrhythmic effect. Despite early evidence suggesting an antiarrhythmic role of n-3 PUFAs in preventing sudden cardiac death and postoperative and persistent AF, subsequent well-designed randomized trials have largely failed to demonstrate an antiarrhythmic benefit. Several trials of moderate- and high-dose n-3 PUFAs showed a reduction in sudden cardiac death, but these results were not widely replicated, and the potential of EPA and DHA in combination or as monotherapy to reduce arrhythmic death remains uncertain [26]. In our present study, co-administration of EPA at 300 mg/kg BW per day dramatically prevented the development of AF by inhibiting HFD-induced inflammation and associated pathophysiological changes. We set the dosage at 300 mg/kg per day to increase the plasma EPA level in mice, which was higher than the usual dosage in humans [17]. However, this EPA dose did not improve chronic HFD-induced systemic metabolic disturbances in mice but did reduce the incidence and duration of AF. This may be beneficial for the treatment of AF in humans.

Although a recent study by da Cunha et al. showed that acute intravenous PUFAs did not directly affect ECG intervals, atrial early repolarization patterns (ERP), or atrial conduction as indicated by P-wave duration [38], Sakabe et al. indicated the chronic beneficial effects of PUFAs on atrial tachycardia remodeling through the oral administration of PUFAs, mimicking dietary PUFA intake that causes gradual PUFA incorporation into cardiac cell membranes [39]. Recent animal studies have shown that statin or peroxisome proliferator-activated receptor-γ activator treatment attenuates AF promotion, at least in part, through anti-inflammatory effects [40]. EPA treatment also resulted in increased levels of adiponectin, an anti-inflammatory adipokine, and decreased levels of TNF-α, a pro-inflammatory adipokine, in the atrium. Taken together, the anti-inflammatory properties of EPA could contribute to the attenuation of AF promotion as a long-term antiarrhythmic effect.

### Study Limitations

This study has several limitations: We did not evaluate direct measurement of ion channel activity using isolated atrial myocytes. So, the precise mechanism of metabolic atrial remodeling in the pathophysiology of high-fat diet-induced AF could not clearly established in this study. The experimental model employed in this study involved the use of murine subjects. It is well-documented that rodents exhibit significant responses to sudden shifts in dietary regimens. Moreover, the cardiac energy metabolism of mice is known to be characterized by a substantial glycolytic component. Thus, it is important to consider the potential limitations of extrapolating the findings from murine models to human subjects. In small animals such as the mouse, the induction of AF is also difficult due to the lack of a critical mass of the atrium [41]. So, it should be noted that the mice utilized in this study do not spontaneously develop AF, thus precluding the possibility of direct extrapolation of results to humans, who do spontaneously develop AF. Actually, sustained AF and/or AF vulnerability can be contributed to the “AF substrates”, which means the one of the factors are imperative to understanding the development of atrial fibrillation (AF) and the process of chronicity. However, sustained AF and/or AF vulnerability has been attributed to the “AF substrates”, which the one of a common causative factor between human and rodents. This finding suggests a potential role for dietary factors in the maintenance of atrial fibrillation, which warrants further investigation.

Meanwhile, we did not evaluate the temporal relationship between the remodeling of ion channels and the development of atrial fibrosis in this study. Although our data suggest that HFD-induced AF is mediated by both ion channel remodeling and atrial fibrosis, it would be challenging to determine which of these two processes occurs first and would require the use of transgenic mouse models of obesity and is therefore beyond the scope of this study. Nevertheless, this is an important question and it is one that we would like to address in future studies.

## Conclusion

This study suggests that EPA supplementation prevents HFD-induced atrial electrocardiographic impairment, which was accompanied by low-grade inflammation of the atrial tissue. Long-term intake of EPA may be a promising nutrient for primary prevention of AF by reducing the increased atrial vulnerability and maintaining normal electrical activity caused by an excess of dietary fat.

## Author contributions

K.H., and M.M. designed research; K.H., K.O., T.S., M.H., N.Z., S.M., and M.M. performed and analyzed experiments; K.H., K.O., and M.M. checked and evaluated electrocardiologic data; K.H., K.O., and M.M. prepared figures, interpreted the results of the experiments, and drafted the manuscripts. All authors have read and agreed to the published version of the manuscript.

## Ethics approval and consent to participate

The study was conducted according to the Kindai University Animal Experimentation Regulations (Approval number: KAAG-2020–015, and KAAG-2023–003) and were carried out according to the guidelines for animal research of the Physiological Society of Japan. Also, this study was conducted according to the guidelines of the Declaration of Helsinki, and were carried out according to the guidelines for animal research of the Physiological Society of Japan to minimize the number of animals used, as well as their suffering.

## Consent for publication

Not applicable.

## Funding

This work was supported by KAKENHI grants #20K11636, #23K10815 to M.M., and the 2023 Kindai University Research Enhancement Grant (IP006) to M.M.

## CRediT authorship contribution statement

**Higashihara Mayo:** Methodology, Investigation, Data curation. **Zaima Nobuhiro:** Methodology, Investigation, Data curation. **Masuda Seiji:** Supervision, Methodology, Investigation, Data curation. **Horii Kosuke:** Writing – review & editing, Writing – original draft, Methodology, Investigation, Data curation, Conceptualization. **Morishima Masaki:** Writing – review & editing, Validation, Supervision, Methodology, Funding acquisition, Formal analysis, Data curation, Conceptualization. **Ono Katsushige:** Writing – review & editing, Supervision, Methodology, Data curation, Conceptualization. **Sumi Tomoko:** Methodology, Investigation, Data curation.

## Declaration of Competing Interest

The authors declare that they have no competing interests.
